# An optimized method to visualize lipid droplets in mouse brain tissue

**DOI:** 10.1016/j.crmeth.2026.101455

**Published:** 2026-05-13

**Authors:** Alicia Rey, Francesco Petrelli, Diana Panfilova, Sofia Madsen, Noéline Héritier, Marlen Knobloch

**Affiliations:** 1 Department of Biomedical Sciences, Faculty of Biology and Medicine, University of Lausanne, 1005 Lausanne, Switzerland

**Keywords:** lipid droplets, brain, neuroscience, lipophilic dyes, BD493/503, perilipin, staining, aging, autofluorescence

## Abstract

Lipid droplets (LDs) are lipid-storage organelles that have gained interest in neurodegenerative diseases, yet their physiological role in the brain is not fully understood. Classical LD detection using lipophilic dyes like BODIPY 493/503 (BD493) or antibodies against LD coat proteins typically reveals few LDs in healthy brain tissue. In contrast, our recently developed endogenous LD-reporter mouse showed numerous LDs in the developing and adult brain without staining. To understand this discrepancy, we examined how tissue preparation and detergent influence LD detection. We found that BD493 performs poorly in brain tissue, whereas other lipophilic dyes allow visualization of many LDs. Antibody-based detection is sensitive to tissue pretreatment but can reveal similar LD numbers as the LD-reporter mouse. We here present an optimized protocol, demonstrating that LDs are abundant in healthy young and aged brains, with larger LDs in aged mice. However, LD detection in aged brains requires caution due to strong LD-like autofluorescence.

## Highlights

• An optimized staining method reveals numerous LDs in the healthy mouse brain
• In brain tissue, BODIPY 493/503 works less well than other lipophilic dyes
• LDs increase significantly in size in aged mouse brains

## Motivation

Existing methods for visualizing lipid droplets (LDs) in mouse brain tissue are limited by poor sensitivity and high background, especially when using classical lipophilic dyes like BODIPY 493/503. This gap has hindered accurate assessment of LD abundance and distribution in healthy and aged brains. To address these challenges, we developed and validated optimized staining approaches using alternative lipophilic dyes and antibody-based detection, enabling reliable visualization of numerous LDs in brain tissue.

## Graphical abstract

**Figure:**
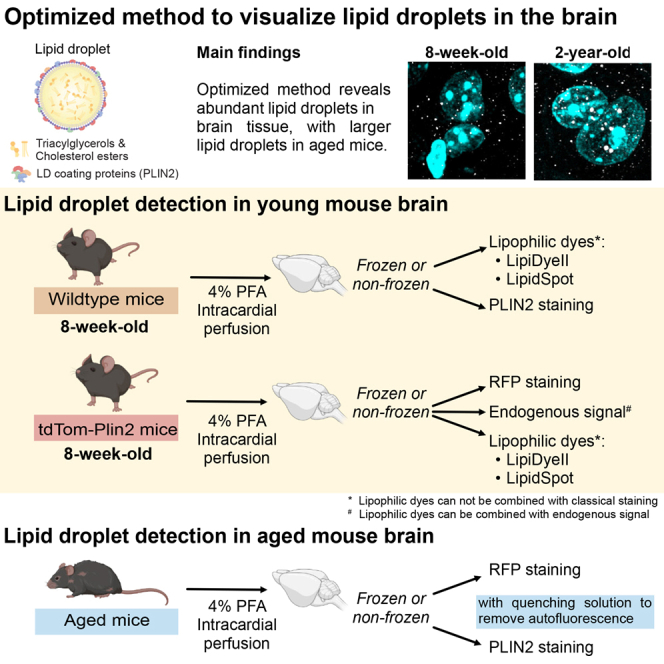

## Teaser

Lipid droplets (LDs) have recently gained interest in neuroscience but visualizing them in the brain is challenging. Rey and Petrelli et al. have optimized LD-staining methods for brain tissue and demonstrate that LDs are numerous in the healthy brain and increase in size with age.

## Introduction

Lipid droplets (LDs) are the lipid storage organelles of cells.^1^^,^^2^ They contain neutral lipids in the form of triacylglycerols (TAGs) and cholesterol esters (CEs), which are surrounded by a phospholipid monolayer. LDs also have various LD coat proteins, such as members of the perilipin family.^1^^,^^2^^,^^3^ Among cell types, adipocytes and hepatocytes have the highest capacity for lipid storage.^4^^,^^5^ Adipocytes, in particular, are specialized cells that can store large amounts of lipids in a single large LD (white adipocytes) or multiple smaller LDs (brown adipocytes).^4^ While these specialized cell types are key to regulate lipid storage, all cell types can form LDs and store neutral lipids to some extent.^6^ LDs were traditionally seen as inert organelles, but recent research has revealed that they are highly dynamic and regulated, with many functions beyond just lipid storage.^6^^,^^7^

LD formation in cells that are not directly involved in lipid metabolism has mainly been associated with disease.^2^ In cases of excess lipid exposure, such as obesity, LDs accumulate ectopically in cells of tissues like the liver, muscle, and heart, resulting in gradual dysfunction and disease.^2^^,^^8^ Another classic example of excessive LD formation is the transformation of macrophages into foam cells, which are filled with LDs containing CEs. These foam cells eventually contribute to the development of atherosclerosis.^9^

Recently, there has been increased interest in LDs in the brain, particularly in relation to neurodegenerative diseases.^10^^,^^11^^,^^12^ Various cell types in the brain, including microglia and astrocytes, have been found to accumulate LDs, particularly in aged mice and in the context of Alzheimer disease.^13^^,^^14^^,^^15^^,^^16^^,^^17^ These LDs appear to play a role in the pathological processes associated with these conditions. Recent studies have also shown that astrocytes can take excess lipids from stressed neurons, store them in LDs, and break them down through fatty acid β-oxidation.^18^ This process may serve as a physiological mechanism for detoxification, and its functioning might be impaired in disease contexts.^18^^,^^19^ These findings have triggered a considerable interest in understanding the role of LDs in both normal brain function and pathology.^10^^,^^11^^,^^12^ However, traditional staining techniques using lipophilic dyes or antibodies against LD coat proteins have only revealed a small number of LDs in healthy brain tissue, aside from the well-known LD-containing ependymal cells.^20^^,^^21^ This raises the question of whether LDs are truly relevant in normal brain physiology, or if there are challenges in detecting them in healthy brain tissue.

We have recently developed an LD-reporter mouse by tagging the endogenous LD protein PLIN2 with tdTomato^22^ (hereafter referred to as tdTom-Plin2). This led to fluorescently labeled PLIN2-positive LDs in all tissues expressing *Plin2*. As tagging proteins can influence their function and stability, we carefully demonstrated that the tagging approach did not alter LD build-up, turnover, and breakdown and that the tdTom-Plin2 knockin mice did not exhibit any histological alterations compared to control mice.^22^ Additionally, we showed that the liver accumulated LDs in a manner similar to control mice when exposed to a high-fat diet.^22^ Surprisingly, we found an abundance of LDs throughout the brain in various cell types in healthy 8-week-old tdTom-Plin2 mice.^22^ LDs were also numerous and highly dynamic in the developing mouse brain. These findings suggest that the issue lies in revealing LDs in the brain, rather than their absence under physiological conditions.

We, therefore, revisited classical LD staining approaches, which are effective in other tissues, and applied them to brain tissue from wild-type (WT) mice. We evaluated the impact of brain tissue freezing on LD detection, compared various commercially available lipophilic dyes, and assessed the influence of tissue permeabilization when utilizing an antibody against PLIN2.

Here, we present our optimized methods for visualizing LDs in healthy mouse brain tissue and provide confirmation of their abundance in the mouse brain. We further demonstrate that revealing LDs in aged brains is challenging due to high autofluorescence that resembles LD-like structure and that commercially available autofluorescence quenchers such as TrueBlack Plus affect the detection of LDs. Despite these challenges, we show that there is a significant accumulation of larger LDs in aged mice.

## Results

### BD493 works in cells but does not reveal LDs in young adult mouse brain tissue

**Figure 1:**
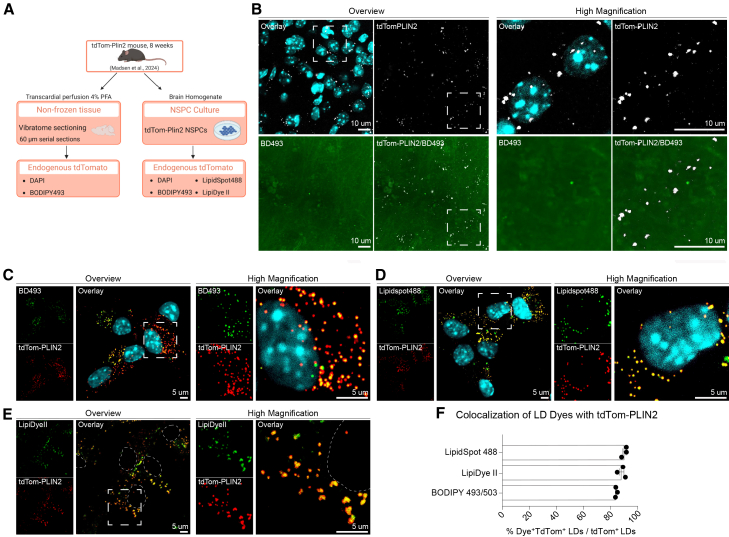
BD493 works in cells but does not reveal LDs in healthy young adult mouse brain tissue (A) Scheme illustrating the processing of the brain tissue and staining procedure used for vibratome-derived sagittal brain sections and for NSPCs from tdTom-Plin2 mice. (B) Representative overview and high-magnification confocal images (maximum projections) showing tdTomato (tdTom-PLIN2, white), DAPI (cyan), and BODIPY493/503 (BD493, green) in the cortex of 8-week-old tdTom-Plin2 mice. Scale bars: 10 μm. (C–E) Representative overview and high-magnification confocal images (maximum projections) showing different lipid dye staining in tdTom-Plin2-derived NSPCs: tdTomato (tdTom-PLIN2, red), BODIPY493 (BD493, green), LipidSpot488 (green), LipiDyeII (green), and DAPI (cyan). Note that due to the excitation of LipiDyeII by 405 nm (Figure S1C), nuclei were not counterstained by DAPI in (E) but are instead outlined with a dotted line. Scale bars: 5 μm. (F) Quantification of co-localized puncta of lipid dyes with tdTomato-PLIN2 in NSPCs derived from tdTom-Plin2 mice. The number of LDs is expressed as a percentage of co-localized puncta between LD dye-positive (LD dye^+^) and tdTom-PLIN2-positive (tdTom^+^) LDs per cell over all tdTom^+^ LDs. Each dot represents data from an individual experiment, with *n* = 3 experiments per group. The data represent the mean value ± SEM. See also Figure S1.

We used brains from 8-week-old tdTom-Plin2 mice (Figure 1A) to either make brain sections for microscopy or extract and culture primary neural stem/progenitor cells (NSPCs). As previously reported, the endogenous tdTom-Plin2 reporter reveals many LDs in the brain of healthy 8-week-old mice.^22^ This is shown here with a representative confocal image of the cortex (Figure 1B) and the subventricular zone (SVZ) (Figure S1A). However, when using the widely used lipophilic dye BODIPY 493/503 (BD493) to detect LDs, we found only a few LDs in the SVZ and almost no LDs in the cortex of tdTom-Plin2 mice (Figures 1B and S1A). This raises questions about whether all the fluorescent tdTomato-positive structures are indeed LDs. Recently, we have shown that primary NSPCs cultured *in vitro* contain a large number of PLIN2-positive LDs^23^ and that the cultured primary NSPCs from tdTom-Plin2 mice also have tdTomato-positive LD-like structures.^22^ Staining of tdTom-Plin2 NSPCs with BD493 revealed a very high co-localization of the two signals (Figure 1C), with ring-like tdTomato-positive structures containing BD493 signal. Quantification showed that around 80% of the tdTomato-positive LDs were also positive for BD493 (Figure 1F), indicating that at least *in vitro*, both the tdTomato and BD493 signals reveal LDs. Interestingly, we consistently detected more LDs with the tdTomato signal than with the BD493 signal, especially smaller structures. There are several potential explanations for this observation: BD493 might require a certain quantity of neutral lipids to label LDs, whereas the LD coat protein PLIN2 is able to reveal smaller LDs as well. Alternatively, this difference could be due to the maturation state of LDs, where tdTom-PLIN2 may also decorate nascent LDs not yet detected by BD493, as has been shown for other perilipins.^24^ Another possibility is that part of the tdTomato signal reflects its degradation. To investigate this, we conducted the co-localization analysis of tdTom-PLIN2 with several markers of the lysosomal/phagosomal pathway. 5%–10% of the tdTomato signal co-localized with lysosomes/autophagosomes, indicating that only a small portion of the signal detected with the endogenously tagged Plin2 comes from its degradation (Figures S1B–S1E).

### Alternative lipophilic dyes detect a large number of LDs *in vitro* and in brain tissue

While BD493 is one of the most commonly used dyes to reveal LDs, there are other lipophilic dyes available commercially that have different chemical properties. We, thus, wanted to compare how these dyes perform in detecting LDs compared with BD493. We chose LipiDyeII, a dye in the green emission spectrum that is designed for live imaging but also works on fixed cells, and a dye class called LipidSpot, which is available in green and far-red (Figures S1F and S1G; of note, far-red is not compatible with tdTomato). These dyes consistently revealed LDs in tdTom-Plin2 NSPCs (Figures 1D and 1E) and showed a similar or slightly higher percentage of co-localization with the tdTomato signal compared with BD493 (Figure 1F), suggesting that they are valid alternatives for LD detection.

**Figure 2:**
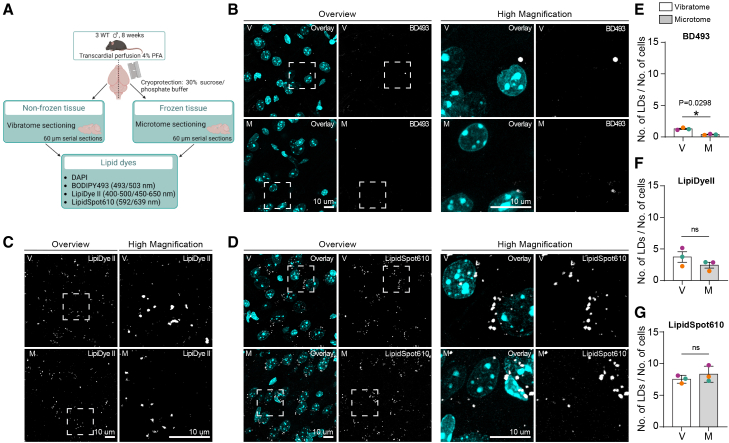
Alternative lipophilic dyes detect a large number of LDs *in vitro* and in brain tissue (A) Scheme illustrating the staining procedure in sagittal brain sections of C57BL/6 (WT) mice, using both microtome (M) and vibratome (V) sectioning techniques. (B–D) Representative overview and high-magnification confocal images (maximum projections) showing different lipid dye staining in M- and V- derived cortical sections of WT mice: BD493 (white), LipiDyeII/LipidSpot610 (white), and DAPI (cyan). Scale bars: 10 μm. (E–G) Graphs showing the quantification of LDs detected with BD493, LipiDyeII, and LipidSpot610, normalized to the number of cells in M- and V-derived cortical sections of WT mice. Each dot represents data from an individual mouse (color coded), with *n* = 3 mice per group. The data present the mean value ± SEM. Paired *t* test, *p* value: ∗, <0.05; ns, non-significant. See also Figure S2.

Next, we assessed and quantified the number of LDs detectable with the different lipophilic dyes in brains from 8-week-old WT mice. After transcardial perfusion with 4% paraformaldehyde (PFA), we used two commonly used tissue processing approaches: vibratome sectioning, which does not require tissue freezing, and microtome sectioning, which is done on frozen tissue that has been incubated in a sucrose solution for cryopreservation (Figure 2A). To compare the influence of the tissue sectioning method, we cut the brains in half and processed each hemisphere in parallel (Figure 2A). As we had observed with the tdTom-Plin2 mouse brain tissue (Figures 1C and S1B), BD493 barely revealed LDs in brain sections from WT mice (Figure 2B). In contrast, LipiDyeII and LipidSpot610 revealed a large number of LDs (Figures 2C and 2D), very similar to what we had observed with the tdTom-Plin2 mouse. Quantification results confirmed that LipiDyeII and LipidSpot610 were far superior to BD493 in detecting LDs (Figures 2E–2G). Tissue sectioning did not significantly affect LD detection, but freezing of the tissue seemed to slightly reduce the numbers of LDs detected for LipiDyeII and BD493, but not for LipidSpot610 (Figures 2E–2G). The background signal was also much lower with LipiDyeII and LipidSpot610 than with BD493 (Figures S2B–S2G). BD493 revealed a remarkable number of fibrous structures, especially in the corpus callosum, which is rich in myelinated fibers (Figure S2E), suggesting that it might also interact with myelin.

Taken together, these results showed that the commonly used lipophilic dye BD493 does not work well for detecting LDs in brain tissue. This might explain why LDs have not been observed in healthy mouse brains by many researchers using BD493. The two alternative dyes performed much better and revealed a large number of LDs. It is worth noting that we were able to successfully stain LDs with BD493 in liver sections using the same staining protocol,^22^ indicating that the issue with BD493 is likely due to tissue-specific properties of brain sections.

### Staining outcome using a PLIN2 antibody in adult mouse brain tissue depends on tissue treatment

**Figure 3:**
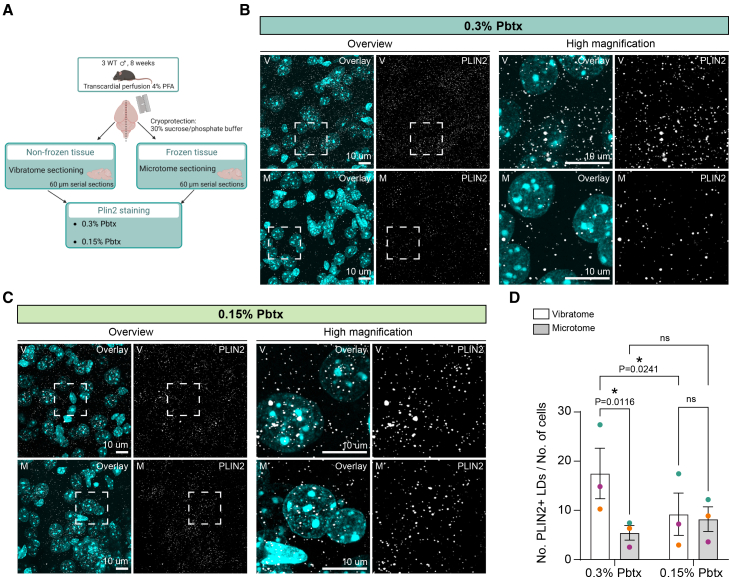
Staining outcome using a PLIN2 antibody in young adult mouse brain tissue depends on tissue treatment (A) Scheme illustrating the PLIN2 immunostaining procedures with two different concentrations of phosphate buffer and Triton (0.15% and 0.3% Pbtx) in sagittal brain sections of C57BL/6 (WT) mice, using both microtome (M) and vibratome (V) sectioning techniques. (B and C) Representative overview and high-magnification confocal images (maximum projections) showing PLIN2 (white) and DAPI (cyan) immunostaining with 0.3% Pbtx (B) and 0.15% Pbtx (C) in M- and V-derived cortical sections of WT mice. Scale bars: 10 μm. (D) Quantification of PLIN2-positive LDs normalized to the number of cells (DAPI), using 0.15% and 0.3% Pbtx in V and M sagittal cortical sections of WT mice. Each dot represents data from an individual mouse (color coded), with *n* = 3 mice per group. The data present the mean value ± SEM. Two-way ANOVA followed by Fisher’s LSD test, *p* value: ∗, <0.05; ns, non-significant. See also Figure S3.

The tdTom-Plin2 knockin reporter mouse enables detection of LDs because of the fluorescent tagging of Plin2, which leads to the expression of tdTom-PLIN2 protein.^22^ This mouse model is a useful tool for studying LDs without the need for staining in both fixed and live tissues and cells. However, it would also be beneficial to be able to visualize LDs using classical immunohistochemistry approaches in brain tissue. Therefore, we optimized the staining parameters by experimenting with different detergents, detergent concentrations, incubation time, and the addition of detergent to primary and secondary antibody solutions. In our experiments, Triton worked best as a detergent when it was added only to the blocking solution and during primary antibody incubation. We compared both vibratome- and microtome-derived sections in parallel, using two Triton concentrations in phosphate buffer, 0.3% and 0.15%, referred to hereafter as 0.3% Pbtx or 0.15% Pbtx (Figure 3A). Overall, we were able to detect a large number of LDs in healthy WT brain sections in all conditions, both in the cortex (Figures 3B and 3C) and in the SVZ, with clear ring-like structures of large LDs in the SVZ (Figures S3A and S3B). Quantification revealed that the highest number of LDs was detected in vibratome-derived sections with 0.3% Triton (Figure 3D). Reducing the detergent concentration to 0.15% also reduced the number of LDs detected (Figure 3D). Additionally, the number of LDs detected was lower in microtome sections regardless of the detergent concentration used (Figure 3D). These findings suggest that treatment of the tissue and the concentration of detergent used can influence the number of LDs that can be detected using immunohistochemistry against PLIN2.

### Endogenous tdTom-PLIN2 signal does not depend on the tissue sectioning method

**Figure 4:**
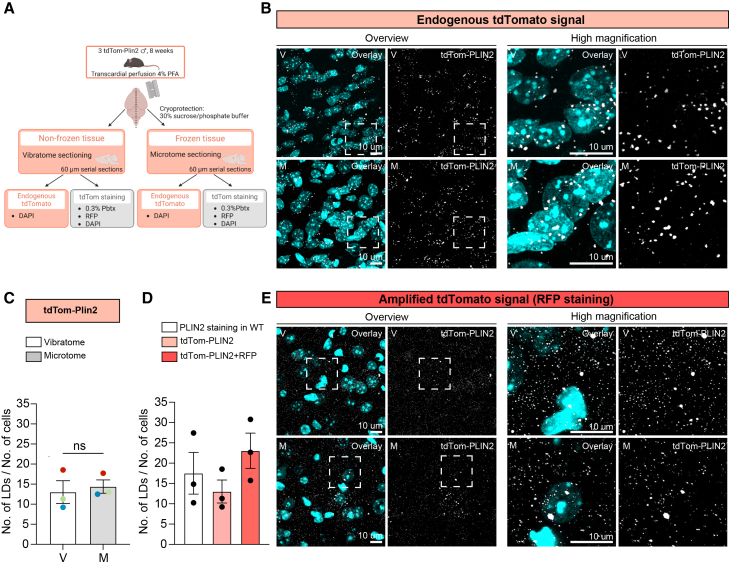
Endogenous tdTom-PLIN2 signal does not depend on the tissue sectioning method (A) Scheme illustrating the experimental procedure in sagittal brain sections of tdTom-Plin2 mice, using both microtome (M) and vibratome (V) sectioning techniques. (B) Representative overview and high-magnification confocal images (maximum projections) showing tdTomato (tdTom-PLIN2, white) and DAPI (cyan) in M- and V-derived cortical sections of tdTom-Plin2 mice. Scale bars: 10 μm. (C) Quantification of tdTom-Plin2-positive LDs normalized to the number of cells (DAPI) in V- and M-derived sagittal brain sections of tdTom-Plin2 mice. Each dot represents data from an individual mouse (color coded), with *n* = 3 mice per group. The data present the mean value ± SEM. Paired Student’s *t* test; ns, non-significant. (D) Quantification of LDs in PLIN2-stained, endogenous tdTom-PLIN2 and tdTom-PLIN2 enhanced with RFP-stained LDs, normalized to the number of cells in WT and tdTom-Plin2 mice. Each dot represents data from an individual mouse, with *n* = 3 mice per group. The data represent the mean value ± SEM. One way ANOVA showed no significance between the groups. (E) Representative overview and high-magnification confocal images depict endogenous tdTom-PLIN2 stained with RFP (white) and DAPI (cyan) in M- and V-derived cortical sections of tdTom-Plin2 mice. Scale bars: 10 μm. See also Figure S4.

To investigate if tissue sectioning affects LD numbers in the endogenous tdTom-Plin2 reporter mouse, we followed the same procedure as with the WT brains (Figure 2A), using brains from 8-week-old tdTom-Plin2 male mice (Figure 4A). Surprisingly, freezing the tissue did not impact the number of LDs detected by tdTomato. LD numbers were similar between microtome and vibratome sections, in both the cortex (Figures 4B and 4C) and the SVZ (Figure S4A). These findings suggested that the treatment of the tissue affects LD detection only when combined with the use of detergents, as observed in WT brain sections (Figure 3D).

### Tissue permeabilization leads to changes in LD size distribution

While the LD numbers in PLIN2-stained WT brains and tdTom-Plin2 brains were comparable (Figure 4D), we noticed slight differences in the LD pattern in WT brain sections (Figures 3B and 3C) and tdTom-Plin2 brain sections (Figures 4B and 4C). Staining for PLIN2 resulted in a higher number of small puncta, and LDs in WT brain sections appeared generally smaller. Interestingly, this pattern also emerged when subjecting the tdTom-Plin2 sections to an immunostaining using an antibody against tdTomato (red fluorescent protein, RFP) (Figures 4D and 4E). To investigate the reasons behind these differences, we quantified the numbers and sizes of LDs under various treatment conditions. We used vibratome and microtome brain sections from tdTom-Plin2 mice and either directly imaged LDs, conducted a “mock” staining (incubating sections with a staining solution that did not contain an antibody), or performed staining with an antibody targeting tdTomato (RFP) to amplify the endogenous tdTomato signal. Interestingly, mock staining resulted in a decrease in overall LDs per cell, while RFP staining increased the total number of LDs detected (Figure S4C). The distribution of LD sizes was also markedly altered by the staining process, as RFP-stained sections exhibited a higher percentage of small LDs and fewer large LDs (Figure S4C), suggesting that the use of detergents can impact the detectable LD size distribution. A similar pattern was observed when staining WT brains against PLIN2, with notably fewer large LDs compared to the endogenous tdTom-Plin2 mouse brains (Figure S4D).

These results suggested that the immunostaining procedures alter the LD size distribution. The cause of this change, whether it is LD shrinkage, lipid leakage during detergent exposure, the inability to detect smaller LDs without immunostaining amplification, or a combination of these factors, remains to be determined.

### Simultaneous detection of the LD core and LD coat works in tdTom-Plin2 brain sections but fails with immunostaining of WT brain sections

**Figure 5:**
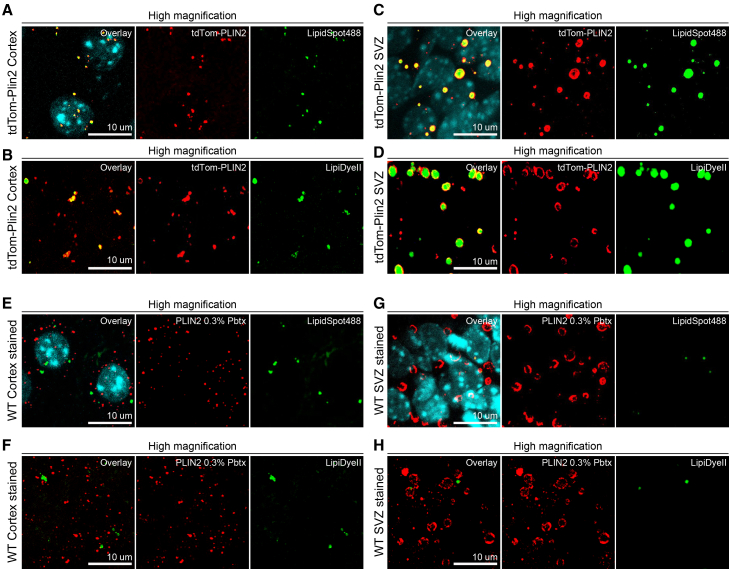
Simultaneous detection of LD core and LD coat works in tdTom-Plin2 brain sections but fails with immunostaining of WT brain sections (A and B) High-magnification confocal images (maximum projections) showing tdTom-PLIN2 (red), LipidSpot488 or LipiDyeII (green), and DAPI (cyan) staining in vibratome-derived cortical sections of tdTom-Plin2 mice. (C and D) High-magnification confocal images (maximum projections) showing tdTom-PLIN2 (red), LipidSpot488 or LipiDyeII (green), and DAPI (cyan) staining in vibratome-derived sections of the SVZ of tdTom-Plin2 mice. (E and F) High-magnification confocal images (maximum projections) showing PLIN2 (red), LipidSpot488 or LipiDyeII (green), and DAPI (cyan) staining in vibratome-derived cortex sections of WT mice. (G and H) High-magnification confocal images (maximum projections) showing PLIN2 (red), LipidSpot488 or LipiDyeII (green), and DAPI (cyan) staining in vibratome-derived SVZ sections of WT mice. Scale bars for all images: 10 μm. See also Figure S5.

Staining of cultured tdTom-Plin2 NSPCs with lipophilic dyes showed clear double labeling, demonstrating that the tdTom-Plin2 construct indeed reports LDs, at least *in vitro* (Figures 1C–1F; also refer to Madsen et al.^22^). Most of the tdTom-Plin2 signal in brain sections is dot-like, but ring-like structures could also be detected in both tdTom-Plin2 sections (Figures 4 and S4; also refer to Madsen et al.^22^) and PLIN2-stained WT brain sections (Figures 3 and S3). To prove that the structures detected in the brain sections are indeed *bona fide* LDs, we co-stained tdTom-Plin2 sections with either the lipophilic dye LipidSpot488 or LipiDyeII. TdTomato and the lipophilic dyes showed clear co-localization in the cortex (Figures 5A 5B, S5A, and S5B), and in larger LDs, a ring-like tdTomato signal surrounded a green lipid dye-labeled core in the SVZ (Figures 5C and 5D), suggesting that these are indeed LDs.

However, when we combined the LipidSpot488 or LipiDyeII staining with immunohistochemistry against PLIN2 in WT brain sections, the lipophilic dyes performed poorly in both cortex and SVZ, despite a clear PLIN2 signal (Figures 5E–5H). This is in strong contrast to the clear signals obtained in WT brain sections when only using the lipophilic dyes without tissue permeabilization (Figures 2C and 2D).

These data suggest that the lipids in LDs might be washed out during the immunohistochemistry process when using brain tissue and that only the LD coat proteins remain. Alternatively, integration of lipophilic dyes in lipid-rich structures might be hindered after the use of detergents. When using the endogenous tdTom-Plin2 LD reporter, there is no need for tissue permeabilization, and co-labeling, therefore, works. However, we noted that the time between tissue sectioning and lipophilic dye staining influenced the outcome in our setting (i.e., sections kept in PBS or cryoprotection solutions at 4°C), and co-labeling no longer worked after the sections were stored for a prolonged time in the solutions. Interestingly, this was not the case for brain tissue that had been kept at 4°C but was not cut into sections. These observations suggest that the lipids might indeed get washed out over time, even without detergents, when the tissue has been sectioned. To enhance the reader’s understanding, we have compiled a diagram summarizing our findings in WT and tdTom-Plin2 brain sections, showing the different staining procedures and their effects (Figure S5C).

### LD-like signal in 2-year-old mice is strongly influenced by autofluorescence

Given the previous literature that LDs accumulate in microglia and astrocytes with aging,^13^^,^^14^^,^^15^^,^^16^^,^^17^ we next examined the cortex of 2-year-old tdTom-Plin2 mice. To our surprise, almost all cells per field of view had substantial accumulation of LD-like fluorescence signal (Figure S6A). To confirm that this extensive LD-like accumulation was not only present in the tdTom-Plin2 reporter mouse but also visible in aged WT mouse brains, we used the optimized staining protocol with an antibody against PLIN2. The cortex of 2-year-old WT mice showed a similar extensive LD-like accumulation in almost all cells per field of view (Figure S6B).

However, when performing a mock staining, during which the primary antibody was omitted, we observed a similar pattern of LD-like fluorescence signal in the red and far-red wavelengths. Of note, very little of this LD-like fluorescence was visible in the green wavelength (Figure S6C). This suggests that the LD-like signal in the aged brains is partially due to autofluorescent structures, which show autofluorescence particularly in the red and far-red channels. This differs from the tissue of young animals, where no autofluorescent signal was detected using the same laser intensity (Figure S6G).

Many substances such as lipofuscin or oxidized lipids can exhibit autofluorescence and are especially prevalent in aged tissue.^25^^,^^26^^,^^27^ We utilized a common autofluorescent quencher called TrueBlack Plus, which is based on the compound Sudan Black B. TrueBlack Plus can be used in PBS but is washed out by detergents and, thus, must be applied at the end of the staining procedure. TrueBlack Plus efficiently eliminated autofluorescence after just 5 min of incubation in unstained sections (Figure S6F). Although the LD-like structures initially observed were significantly reduced after the TrueBlack Plus treatment, we were still able to reveal many LDs in both WT sections stained against PLIN2 and tdTom-Plin2 brain sections stained against RFP of 2-year-old mice (Figures S6D and 6E). The specificity of PLIN2 staining was confirmed through a mock staining using only the secondary antibody and a 5-min incubation with TrueBlack Plus on WT tissue where no signal was detected in any of the three channels (Figure S6F).

### LDs in the brains of 2-year-old mice are larger than in young mice

To accurately compare the number of LDs between young and aged mice, all sections should undergo the same staining procedure. While aged brain tissue exhibited a significant amount of LD-like autofluorescent signal, necessitating quenching (Figure S6C), young brain sections did not show autofluorescence under the same microscopy settings (Figure S6G). Therefore, we first assessed whether treating young brain sections with TrueBlack Plus would affect the detection of LDs.

Interestingly, brain sections from 8-week-old WT mice stained against PLIN2 showed a significant reduction in the number and total volume of detected LDs after a 5-min treatment with TrueBlack Plus, compared with sections that were not treated with TrueBlack Plus (Figures S6H–S6K). As TrueBlack Plus was applied at the end of the staining procedure due to its incompatibility with detergents, the decrease in LDs suggests that TrueBlack Plus also partially suppresses the fluorescence of the secondary antibody, resulting in fewer LDs being detected. This likely occurs in the aged tissue as well, and, thus, the total amount of LDs may be underestimated using this method.

**Figure 6:**
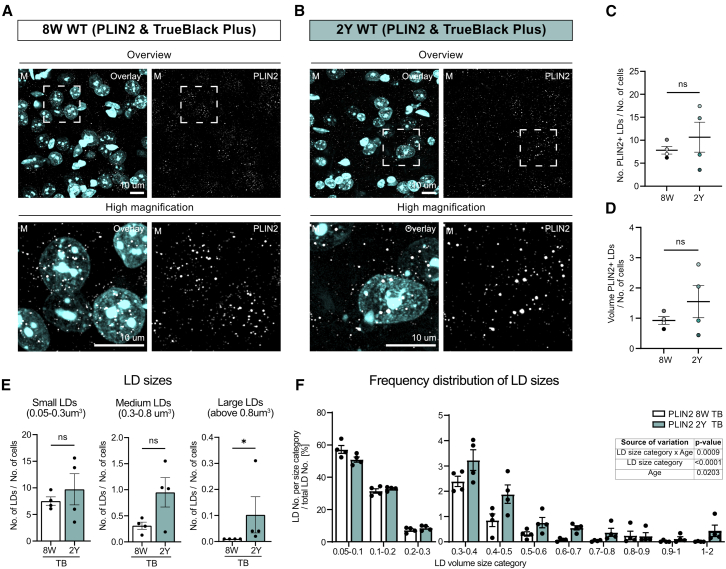
The number of LDs in the brains of 2-year-old mice is variable with larger sizes compared to young mice (A) Overview and high-magnification confocal images (maximum projections) showing PLIN2 (white) and DAPI (cyan) in the cortex of a 8-week-old WT mouse after 5-min treatment with TrueBlack Plus. Scale bars: 10 μm. (B) Overview and high-magnification confocal images (maximum projections) showing PLIN2 (white) and DAPI (cyan) in microtome sections of the cortex of 2-year-old WT mouse after 5-min treatment with TrueBlack Plus. Scale bars: 10 μm. (C) Quantification of the total number of PLIN2-positive LDs per number of cell at 8 weeks and 2 years. Each dot represents an individual mouse, with *n* = 4 mice per group. The data present the mean value ± SEM. Unpaired Student’s *t* test; ns, non-significant. (D) Quantification of the total volume of PLIN2-positive LDs per number of cell at 8 weeks and 2 years. Each dot represents an individual mouse, with *n* = 4 mice per group. The data present the mean value ± SEM. Unpaired Student’s *t* test; ns, non-significant. (E) Quantification of the number of small-sized LDs (0.05–0.3 μm^3^), medium-sized LDs (0.3–0.8 μm^3^), and larger-sized LDs (above 0.8 μm^3^) in WT cortex sections from 8-week-old and 2-year-old mice after 5-min treatment with TrueBlack Plus. The data represent the mean value ± SEM. Unpaired Student’s *t* test; *p*-value: ∗ < 0.05; ns, non-significant. (F) Bar graphs showing the total number of larger LDs and their respective size frequency distribution, ranging from 0.3 μm^3^ to above 1 μm^3^. Each dot represents an individual mouse, with *n* = 4 mice per group. Two-way ANOVA (factors: age and LD size distribution) after transformation of data using arcsin(sqrt(Y)). Effects of age (*p* value = 0.0203), LD size distribution (*p* value < 0.0001), and age × LD size distribution interaction (*p* value = 0.0009). The data representing the mean value ± SEM are depicted for the total number of LDs. See also Figure S6.

We proceeded to analyze if there is a difference in the number of LDs in the cortex of 8-week-old and 2-year-old WT mice, using staining against PLIN2, followed by a 5-min incubation with TrueBlack Plus (Figures 6A and 6B). LD numbers and total volume were higher in the 2-year-old mice, but this difference did not reach statistical significance due to a large variability between animals in the 2-year-old group, which was not observed in the 8-week-old mice (Figures 6C and 6D).

Nevertheless, when comparing the number of LDs with different size categories, there was a significant shift toward larger LDs in the 2-year-old mice compared to the 8-week-old mice (Figure 6E). This became even more evident when plotting the percentage of each size category over the total number of LDs: statistical analysis showed a significant effect of age and a significant interaction of age and LD size (Figure 6F). Together, these data show that LD numbers tend to be higher and significantly increased in size in aged mice but that there is a large inter-animal variability.

## Discussion

LDs in the brain have gained significant interest in recent years, as they appear to be involved in normal brain function and may also be directly implicated in several neurological diseases.^2^^,^^10^^,^^11^^,^^12^ However, due to their unique structure, consisting of a phospholipid monolayer and a lipid-rich core, LDs are fragile and tissue processing and detergents used for immunohistochemistry are likely to influence their detection.^28^^,^^29^ Therefore, caution must be exercised when selecting a method to visualize LDs, particularly when making statements about their absence, as this could be due to the chosen detection method. Several established techniques exist for detecting LDs, including the use of various lipophilic dyes^30^ and immunohistochemistry targeting LD coat proteins.^31^ Additionally, several advanced microscopy techniques that exploit the specific properties of lipids, such as high optical diffraction or specific vibrational characteristics, have been developed for label-free LD detection.^32^^,^^33^^,^^34^ However, these microscopy techniques require specialized equipment, which make them less accessible. While staining methods work well in cultured cells, detecting LDs in tissues with these methods is more challenging. The brain is a lipid-rich tissue, with lipids accounting for over 50% of its dry weight,^35^^,^^36^ and the detection of LDs with lipophilic dyes appears to differ from that in other tissues. Here, we demonstrated that the commonly used dye BD493, which performs effectively in cells and various tissues like the liver, does not yield satisfactory results in brain sections (Figure 1). It exhibits a low signal-to-noise ratio and significant background signal. Surprisingly, other lipophilic dyes with distinct chemical structures, such as LipiDyeII, LipidSpot488, and LipidSpot610, performed much better in brain sections compared to BD493 (Figure 2). We confirmed that all dyes performed similarly well in cultured NSPCs *in vitro*, ruling out a general issue with our BD493 protocol (Figure 1). The reason for this discrepancy in dye performance is unclear, but it underscores the significant impact that the choice of lipophilic dye can have on results. Tissue penetrance and binding properties may vary among the dyes. Given the lipid-rich nature of brain tissue, certain lipophilic dyes may have a greater tendency to interact with myelin, potentially reducing their accumulation in LDs. Indeed, we observed clear fiber tracks when using BD493, especially in the corpus callosum, which is rich in myelinated fibers (Figure S2). Interestingly, recent studies have revealed that the presence of crystalline or liquid cores in LDs depends on the ratio of TAGs to CEs.^37^ Whether this phenomenon influences LD detection in the brain, and whether different lipophilic dyes perform differently depending on the core structure remain to be determined. Different lipid species in the LD core might also influence binding properties of the different dyes; thus, we suggest using several different lipophilic dyes when studying LDs in the brain.

One advantage of lipophilic dyes is that they do not require the use of detergents, which can impact the detection of LDs. However, because lipophilic dyes do not target specific structures and, instead, accumulate in lipid-rich compartments, they may also accumulate in structures other than LDs. Indeed, a recent publication demonstrated that Nile red, a commonly used lipophilic dye, accumulated in non-LD compartments.^38^ Thus, a combination of different detection methods for LDs in the brain is advisable.

As LDs are rather fragile organelles, tissue treatment is likely to influence their detection. We maintained the initial fixation of the tissue using 4% PFA. However, it remains to be investigated whether different tissue fixation methods also affect LD detection. In this study, we evaluated two parameters of tissue treatment: freezing or not freezing the tissue for sectioning, and the percentage of Triton used as a detergent during immunohistochemistry (Figure 2). To prevent water crystal formation during the freezing process, we utilized the well-established cryopreservation strategy of immersing the brain in 30% sucrose solution before freezing. The tdTom-Plin2 mouse^22^ allowed us to distinguish between the effects of tissue treatment and detergent concentration, as the endogenous fluorescent LDs were detected without the need for immunohistochemistry. Interestingly, freezing or not freezing the tissue prior to cutting influenced LD detection only when immunohistochemistry was performed (Figure 3), but no significant difference was observed in the tdTom-Plin2 sections (Figure 4). This suggests that LDs are not inherently destroyed by tissue freezing processes, but the subsequent use of detergent can affect LD detection. Lipids, which are notably not crosslinked by PFA and, thus, not fixed, may leak out and alter LD detection. Using the tdTom-Plin2 sections and performing either a mock staining or staining against RFP showed that the LD size distribution and the total number of LDs detectable changed (Figure S4), supporting the hypothesis that lipids may leak out with detergent use. This is also in line with our findings that the LD coat proteins were more consistently detected than the LD core (Figure 5). Additionally, the simultaneous detection of LD coat and LD core was achievable only with the tdTom-Plin2 reporter mouse, where no detergents were required to reveal the LD coat protein. However, in WT mice, simultaneous detection of LD coat and LD core was not possible, likely due to lipid leakage after the immunochemistry procedure (Figures 5 and S5). In line with this, we also observed less reliable lipophilic dye staining with larger LDs, such as those found in the SVZ, suggesting that lipids from larger LDs may be more prone to leakage. We also noted that when using lipophilic dyes, it is important to proceed to the microscopy within one week, as the dyes can leak even when the tissue or cells are mounted on glass carriers and embedded with antifade solution.

While our data clearly demonstrate that LDs are also abundant in the healthy brain and at young age, the specific role they play is largely unknown. Using the tdTom-Plin2 LD reporter mouse, we have shown that various types of brain cells contain LDs varying in the number and size.^22^ These findings suggest that having a certain number of LDs may be physiologically normal. Indeed, the recent discovery that astrocytes take up excessive lipids from neurons and store them in LDs^18^ highlights the close metabolic interplay between these two cell types. However, we are only beginning to understand the physiological function of LDs in the brain. Therefore, further research is necessary to uncover their dynamics and to understand their importance for normal brain function.

Our protocol further highlights that the visualization of LDs in aged brain tissue is particularly challenging due to the presence of highly autofluorescent structures in the old brain tissue, which often appear as punctate LD-like structures. The use of the commercially available quencher TrueBlack Plus substantially reduced this autofluorescence; however, it also partially quenches secondary antibody fluorescence, thereby reducing the numbers of LDs that could be detected using this approach. Therefore, the actual number of LDs in the aged mice might be underestimated, and additional approaches are needed to determine the extent of LD accumulation in the aged brain.

Autofluorescence in aged tissue has been mainly attributed to lipofuscin, a heterogeneous mixture of partially degraded proteins and lipids that accumulate with age.^25^^,^^26^ Lipofuscin exhibits strong, broad-spectrum punctate autofluorescence, which interferes with immunohistochemical analyses in which punctate structures are part of the specific readout, for instance, in microglial engulfment analyses.^27^ In this context, the use of quenchers that physically associate with autofluorescent aggregates and absorb their emission is useful. However, as oxidized lipids also exhibit intrinsic autofluorescence, such quenching approaches might also attenuate LD-related signals. Other strategies such as photobleaching using high-power LED illumination might be an alternative to chemical quenching.^39^

LD accumulation in the aged brain has been previously reported.^13^^,^^14^^,^^15^^,^^16^^,^^17^ In line with this literature, we saw a significant increase in the number of large LDs in the brains of 2-year-old WT mice. Interestingly, there is a substantial inter-individual variability in LD accumulation at this age, which is not observed in 8-week-old mice. The underlying mechanisms driving this variability are not yet known, but they may be relevant for age-related diseases. Furthermore, it is unclear whether there are functional consequences of increased LD accumulation, and whether this affects specific cell types or is, instead, a global aging effect. These are questions that need to be addressed in future studies.

An overall increase in LDs in the brain is also supported by several lipidomics studies in mouse brains of varying ages.^40^^,^^41^ While these studies did not specifically focus on LDs, they did report a significant increase in TAGs with aging, which might be linked to a more global metabolic change in the brain with age. Further studies are required to study why LDs accumulate and how this accumulation affects cellular functionality. Furthermore, it will be interesting to study whether there are regional and temporal differences in the accumulation of LDs with age.

Taken together, our data show that LDs are abundant in the healthy adult mouse brain and increase in size with aging. LDs can be detected using our endogenous tdTom-Plin2 reporter line,^22^ as well as lipophilic dyes or immunohistochemistry. We show that tissue treatment, selection of dyes, and detergent concentration all have a clear influence on the detection of LDs. Therefore, care must be taken, and researchers should utilize several methods to assess LDs in the brain. Our results provide a basis for other researchers interested in studying LDs in the brain, helping them choose an appropriate staining method. As LDs have become an important topic in the field of neurodegenerative diseases, it is also important to highlight the challenges in visualizing LDs in the aged brain due to the substantial LD-like autofluorescence signal and the caveats of using chemical quenchers.

### Limitations of the study

While we have addressed several parameters, such as tissue processing and detergent concentrations, we have always used 4% PFA-fixed brain tissue as the starting material. Therefore, we could not determine whether the optimized method will also work with different starting materials, such as, for instance, snap-frozen brain tissue. We also controlled the time the tissue was in fixative (overnight post-fixation after transcardial perfusion); thus, it remains to be determined if prolonged tissue fixation might influence LD detection. For the immunohistochemistry part, our focus was on the LD coat protein PLIN2, given that our endogenous LD reporter mouse is based on the expression of PLIN2. However, there are many other LD coat proteins that could also be used as an antigen target to reveal LDs in brain tissue. Additionally, we found that lipophilic dyes differ in their abilities to detect LDs in brain tissue. We selected a few commercially available ones, but other dyes may also work equally well. The chemical autofluorescence quencher TrueBlack Plus solves the autofluorescence problem but also affects LD detection, leading to an underestimation of LDs. Other options of addressing autofluorescence that might be better suited in this specific case need to be explored.

## Resource availability

### Lead contact

Requests for further information and resources should be directed to and will be fulfilled by the lead contact, Marlen Knobloch (marlen.knobloch@unil.ch).

### Materials availability

This study did not generate new unique reagents.

### Data and code availability

• All data reported in this paper will be shared by the lead contact upon request.
• This paper does not report original code.
• Any additional information required to reanalyze the data reported in this paper is available from the lead contact upon request.

## Acknowledgments

We thank the Cellular Imaging Facility and the Animal Facility of the University of Lausanne for technical support and Frédéric Schütz (UNIL) for statistical advice. This work was supported by funding from the 10.13039/501100006390University of Lausanne and the 10.13039/501100001711Swiss National Science Foundation (grant # 31003A_175570, to M.K.).

## Author contributions

F.P. and A.R. performed experiments and analyzed and visualized the data; D.P. performed the experiments in NSPCs; S.M. and M.K. developed the tdTom-Plin2 mouse model; N.H. performed experiments and analyzed data; A.R., F.P., and M.K. developed the concept and wrote the manuscript, with input from all authors; and M.K. provided the financial means to execute this project.

## Declaration of interests

The authors declare no competing interests.

## Declaration of generative AI and AI-assisted technologies in the writing process

During the preparation of this work, the authors used the free editing option of the Service “Edit my English” (https://www.editmyenglish.com) in order to improve the text. After using this tool, the authors reviewed and edited the content as needed and take full responsibility for the content of the publication.

## STAR★Methods

### Key resources table

**Table:** 

REAGENT or RESOURCE	SOURCE	IDENTIFIER
**Antibodies**		

Rabbit Polyclonal Perilipin-2 antibody	Abcam	Cat# ab52356; RRID: AB_2223599
Rabbit Anti-RFP Polyclonal Antibody, Unconjugated	Abcam	Cat# ab62341; RRID: AB_945213
Rabbit Anti-IBA1	FUJIFILM Wako Pure Chemical Corporation	Cat# 019–19741; RRID: AB_839504
Rabbit Anti-RFP Polyclonal antibody	Abcam	Cat# AB62341; RRID: AB_945213
Goat Anti-tdTomato Polyclonal antibody, unconjugated	Sicgen Antibodies	Cat# AB8181; RRID: AB_2722750
Mouse Anti-NeuN Antibody, clone A60	Sigma-Aldrich	Cat# MAB377; RRID: AB_2298772
Mouse monoclonal Anti-S-100 (beta-Subunit) antibody produced	Sigma-Aldrich	Cat# S2532; RRID: AB_477499
Rabbit Anti-Glial Fibrillary Acidic Protein antibody produced	Sigma-Aldrich	Cat# G9269; RRID: AB_477035
Rabbit Anti-OLIG2 Polyclonal antibody	Millipore	Cat# AB9610; RRID: AB_570666
Rabbit Anti-LAMP1 antibody - Lysosome Marker	Abcam	Cat# AB24170; RRID: AB_775978
Rabbit Anti-Rab7 Monoclonal, clone D95F2	Cell Signaling Technology	Cat# 9367; RRID: AB_1904103
Rabbit Anti-MAP1LC3B Polyclonal antibody	Sigma Aldrich	Cat# L7543; RRID: AB_796155
Alexa Fluor 488-AffiniPure Donkey Anti-Rabbit IgG (H + L)	Jackson ImmunoResearch Labs	Cat# 711-545-152; RRID: AB_2313584
Alexa Fluor® 647 AffiniPure Donkey Anti-Mouse IgG (H + L)	Jackson ImmunoResearch Labs	Cat# 715-605-150; RRID: AB_2340862
Alexa Fluor 647-AffiniPure Donkey Anti-Rabbit IgG (H + L) (min X Bov,Ck,Gt,GP,Sy Hms,Hrs,Hu,Ms,Rat,Shp Sr Prot)	Jackson ImmunoResearch Labs	Cat# 711-605-152; RRID: AB_2492288
Alexa Fluor® 594 AffiniPure Donkey Anti-Rabbit IgG (H + L)	Jackson ImmunoResearch Labs	Cat# 711-585-152; RRID: AB_2340621
Alexa Fluor 594-AffiniPure Donkey Anti-Goat IgG (H + L) (min X Ck,GP,Sy Hms,Hrs,Hu,Ms,Rb,Rat Sr Prot)	Jackson ImmunoResearch Labs	Cat# 705-585-147; RRID: AB_2340433

**Chemicals, peptides, and recombinant proteins**		

BODIPY™ 493/503 (4,4-Difluoro-1,3,5,7,8-Pentamethyl-4-Bora-3a,4a-Diaza-*s*-Indacene)	Fisher scientific	Cat# 11540326
LipiDye II, Lipid dye Droplet Staining	Anawa	Cat# FDV-0027
Lipidspot488 - LipidSpot™ Lipid Droplet Stains	Biotium	Cat# CAT 70065-T
Lipidspot610- LipidSpot™ Lipid Droplet Stains	Biotium	Cat# 70069-T
4′, 6-diamidino-2-phenylindole (DAPI)	Sigma-Aldrich	Cat# D9542-1MG; RRID: SCR_013672
Glycerol, BioUltra, for molecular biology, anhydrous, ≥99.5% (GC)	Sigma-Aldrich	Cat# 49782-1L
Poly(vinyl alcohol) – PVA 87–90% hydrolyzed, average mol wt 30,000–70,000	Sigma-Aldrich	Cat# P8136
Diazabicyclo[2.2.2]octane (DABCO)	Sigma-Aldrich	Cat# D27802-100G
FluorSave™ reagent	Sigma-Aldrich	Cat# 345789
Triton X-100	Sigma-Aldrich	Cat# X100-100ML
TrueBlack Plus	Biotium	Cat# 23014
Donkey serum	Merck	Cat# S30-100mL
Ethylene glycol	Sigma-Aldrich	Cat# 324558-1L
Sodium azide solution (NaN3 0.05%)	Sigma-Aldrich	Cat# RTC000068
Paraformaldehyde, EM Grade, Purified	Electron microscopy	Cat# 19208; CAS #30525-89-4
DMEM/F-12, GlutaMAX™ supplement	Gibco	Cat# 31331-028
B-27™ Supplement (50X), serum free	Gibco	Cat# 17504044
*N*-2 Supplement (100x)	Gibco	Cat# 17502048
Human EGF, Animal-Free Recombinant Protein	PeproTech	Cat# AF-100-15
Human FGF-basic (FGF-2/bFGF) (154 aa) Recombinant Protein	PeproTech	Cat# 100-18B
Heparin sodium salt from porcine intestinal mucosa	Sigma-Aldrich	Cat# H3149-50KU
Poly-L-ornithine hydrobromide	Sigma-Aldrich	Cat# P3655
Mouse Laminin from Engelbreth-Holm-Swarm murine sarcoma basement membrane	Sigma-Aldrich	Cat# L2020-1MG
FluorSave™ Reagent	Merck Millipore	Cat# 345789
Antibiotic-Antimycotic (100X)	Gibco	Cat# 15240062
Glycine	Sigma-Aldrich	Cat# G8898
BSA	Sigma-Aldrich	Cat# A8806-5G
Saponin	Sigma-Aldrich	Cat# 84510-100G

**Critical commercial assays**		

MACS Neural Tissue Dissociation Kit (Papain-based)	Miltenyi Biotec	RRID: SCR_020293; Cat# 130-092-628
MACS Myelin Removal Beads II	Miltenyi Biotec	Cat# 130-096-731
QuadroMACS Separator	Miltenyi Biotec	Cat# 130-090-976
gentleMACS Dissociator	Miltenyi Biotec	Cat# 130-093-235

**Experimental models: Cell lines**		

Neural progenitor stem cells extracted from C57BL/6Rj WT mouse line	Janvier	N/A
Neural progenitor stem cells extracted from tdTom-Plin2 mouse line	Prof. Marlen Knobloch (University of Lausanne, Lausanne)	N/A

**Experimental models: Organisms/strains**		

Mouse: C57BL/6Rj WT	Janvier Labs	N/A
Mouse: tdTom-Plin2 mouse line	Prof. Marlen Knobloch (University of Lausanne, Lausanne)	N/A

**Software and algorithms**		

Imaris	http://www.bitplane.com/imaris/imaris	RRID: SCR_007370
ImageJ	https://imagej.net/	RRID: SCR_003070
ilastik	http://ilastik.org/	RRID: SCR_015246
GraphPad Prism	https://www.graphpad.com/	RRID: SCR_002798

**Other**		

Cell culture dishes, TC-treated	Corning	Cat# 430167
Glass coverslips	Fisher Scientific	Cat# 12-545-80
Superfrost Plus microscope slides	Epredia	Cat# J1800AMNZ

### Experimental model and study participant details

#### Animals

All experiments involving animals were conducted in accordance with the Swiss law and received prior approval from the local authorities (Cantonal Veterinary office, Vaud, Switzerland). TdTom-Plin2 mice were generated as described.^22^ 8-week-old and 2-year-old tdTom-Plin2 mice and C57BL/6Rj WT mice (Janvier, France) were used in this study. All mice were kept under standard housing on a 12:12 h light/dark cycle, in ventilated cages with *ad libitum* food and water. Male mice were used for all experiments, except for NSPC extraction. For the detailed comparison of LD accumulation in different cell types, 2 males and 2 females were used for the 8-week-old tdTom-Plin2 mice and 3 females and 1 male were used for the aged 2-year-old tdTom-Plin2 mice, due to the limited availability of old tdTom-Plin2 mice.

#### NSPC extraction and expansion

Adult mouse NSPCs were isolated from the SVZ of two 8-week-old tdTom-Plin2 female mice as previously described.^42^ In brief, mice were shortly anesthetized with isoflurane, followed by decapitation. SVZ were micro-dissected, and a single cell suspension was generated using the papain-based MACS Neural Tissue Dissociation Kit (#130-092-628, Milteny) and the GentleMacs Dissociator (Milteny), according to the manufacturer’s instructions. Myelin removal was performed using the MACs myelin removal beads (#130-096-731, Milteny) and a QuadroMACS Separator (#130-090-976, Milteny) according to the manufacturer’s instructions. The obtained cells were cultured as neurospheres in DMEM/F12/GlutaMAX (#31331-028, Gibco) with B27 (#17504044, Gibco), 20 ng/mL human EGF (#AF-100-15, PeproTech), 20 ng/mL human basic FGF-2 (#100-18B, PeproTech), and 1x PSF (#15240062, Gibco). Medium was changed every 2–3 days. The neurospheres were expanded for 5 passages to remove progenitors and other proliferating cells. After 5 passages, cells were changed to the following culture medium: DMEM/F12/GlutaMAX (#31331-028, Gibco), N2 (#17502048, Gibco), 20 ng/mL human EGF (#AF-100-15, PeproTech), 20 ng/mL human basic FGF-2 (#100-18B, PeproTech), 5mg/ml Heparin (#H3149-50KU, Sigma) and 1x PSF (#15240062, Gibco). All the *in vitro* experiments were done on passages 7–15.

### Method details

#### Cell culture

NSPCs were grown on uncoated plastic cell culture dishes for expansion (#430167, Corning, TC-treated). Cells used for experiments were plated on glass coverslips (#10337423, Fisher) coated with poly-L-ornithine (#P3655, Sigma) and laminin (#L2020-1MG, Sigma). Proliferating NSPCs were kept in DMEM/F12/GlutaMAX (#31331-028, Gibco) complemented with N2 (#11520536, Gibco), 20 ng/mL human EGF (#AF-100-15, PeproTech), 20 ng/mL human basic FGF-2 (#100-18B, PeproTech), 5 mg/mL Heparin (#H3149-50KU, Sigma) and 1X PSF (#15240062, Gibco). Medium was changed every 2–3 days.

#### Tissue preparation

Perfusion: All experimental mice were deeply anesthetized through intraperitoneal injection (i.p.) of pentobarbital (150mg/kg) and subsequently intracardially perfused first with ice-cold 0.9% saline until no blood remained, then perfused with 40 mL fresh 4% paraformaldehyde (PFA, #19208, Electron Microscopy Sciences, EMS) in 0.1M phosphate-buffered saline (pH 7.4). The brains were post-fixed overnight at 4°C in PFA. After post-fixation, the brains were divided in two hemispheres and stored at 4°C in PBS supplemented with 0.02% sodium azide (RTC000068, Sigma).

Half of the hemispheres were cut in sagittal sections of 60μm using a vibratome (Leica) and stored at 4°C in 1X PBS supplemented with 0.02% sodium azide (#RTC000068, Sigma Aldrich). The other hemispheres were incubated in sucrose 30% in phosphate 0.1M at 4°C for 48 h for cryoprotection. They were then frozen on a dry ice-cooled metal stage and cut in 60μm sagittal sections on a sliding microtome (Leica). The sections were stored in cryopreservation solution (25% ethylene glycol (#324558-1L, Sigma-Aldrich), 25% glycerol (#49782-1L, Sigma-Aldrich) in 0.05 M phosphate buffer) at 4°C.

#### Immunocytochemistry

For the immunocytochemistry analysis of NSCPs, cells were fixed with 4%PFA (pre-warmed at 37°C) for 20 min at RT, followed by two washes with 1X PBS for 10 min. Subsequently, the fixed cells were blocked for 45 min in blocking buffer (1.5% Glycine, 3% BSA, 0.01% Saponin in 1X PBS) and then immunolabeled over night at 4°C without agitation in antibody diluent (0.1% BSA, 0.01% Saponin in 1X PBS) with following primary antibodies: rabbit-LAMP1 (1:500, #ab24170, Abcam), rabbit-Rab7 (1:100, #9367, Cell Signaling Technology) or rabbit-LC3B (1:500, #L7543, Sigma). After the primary antibody incubation, the cells were warmed up to RT for 30 min, washed three times in 1X PBS for 10 min and incubated for 1 h protected from light at RT with fluorescent secondary antibody (1:250, AlexaFluor, anti-rabbit 488, Jackson, 711-545-152) diluted in antibody diluent. Then, cells were washed one time in 1X PBS for 10 min followed by nuclei staining with 4′, 6-diamidino-2-phenylindole (DAPI) (Invitrogen, 1:5000) for 10 min in 1X TBS. Finally, cells were washed one time in 1X TBS for 10 min and mounted on 25 × 75 × 1 mm Superfrost microscope slides (#J1800AMNZ, Epredia) using homemade PVA-DABCO-based mounting medium.

#### Immunohistochemistry

For PLIN2 immunohistochemical analysis, sections were washed three times in 1X PBS for 5 min on orbital shaker at RT. Sections were permeabilized for 1 h in 1X PBS containing 0.3% or 0.15% Triton X-100 (#X100-100ML, Sigma), and 10% donkey serum (#S30-100mL, Merck) and then immunolabeled for 48 h at 4°C on an orbital shaker, using 5% donkey serum (#S30-100mL, Merck) and the following primary antibodies: Rabbit-PLIN2 (1:600, #ab52356, Abcam), rabbit-RFP (1:600, ab62341, Abcam). After the primary antibody incubation, the sections were washed again three times in 1X PBS for 10 min and incubated for 2 h at RT with fluorescent secondary antibodies (1:300, AlexaFluor, anti-rabbit 488, donkey anti-rabbit 594, Jackson, 711-545-152) diluted in 1X PBS. Finally, nuclei were counterstained with 4′, 6-diamidino-2-phenylindole (DAPI) (Invitrogen, 1:10000) for 15 min in 1X PBS, washed two times in 1X PBS. Sections were mounted on 25 × 75 × 1 mm Superfrost Plus adhesion microscopes slides (#J1800AMNZ, Epredia) using FluorSave reagent (#345789, Sigma).

For immunohistochemical analysis using the autofluorescent quencher called TrueBlack Plus (#23014, Biotium), sections were washed three times in 1X PBS for 5 min on orbital shaker at RT 10% donkey serum (#S30-100mL, Merck) and then immunolabeled for 48 h at 4°C on an orbital shaker, using 5% donkey serum (#S30-100mL, Merck) and the following primary antibodies: Rabbit-PLIN2 (1:600, #ab52356, Abcam), rabbit-RFP (1:600, ab62341, Abcam). After the primary antibody incubation, the sections were washed again three times in 1X PBS for 10 min and incubated for 2 h at RT with fluorescent secondary antibodies (1:300, AlexaFluor, anti-rabbit 488, donkey anti-rabbit 594, Jackson, 711-545-152) diluted in 1X PBS. Finally, nuclei were counterstained with 4′, 6-diamidino-2-phenylindole (DAPI) (Invitrogen, 1:10000) for 15 min in 1X PBS, washed two times in 1X PBS. TrueBlack Plus was diluted 1:40 with 1XPBS and incubated for 5, 10 and 15 min, washed two times in 1X PBS. Sections were mounted on 25 × 75 × 1mm Superfrost Plus adhesion microscopes slides (#J1800AMNZ, Epredia) using FluorSave reagent (#345789, Sigma).

#### Lipophilic dye staining in NSPCs and brain tissue

LDs in NSPCs were detected as following: Cells were fixed with 4%PFA (pre-warmed at 37°C) for 20 min at RT, followed by two washes with 1X PBS for 10 min. Subsequently, the fixed cells were stained for 1 h at RT with the following lipophilic dyes, diluted in 1X PBS: BODIPY 493/503 4,4-DIFLUORO-1,3,5,7,8-PE (11540326, Fisher Scientific) (1:1000), LipidSpot 488 or 610 (VWR 70065-T or 70069-T) (1:1000), LipiDyeII (Anawa, FDV-0027) (1:1000). Cells were subsequently washed three times in 1X PBS for 5 min. After that, nuclei were counterstained with 4′, 6-diamidino-2-phenylindole (DAPI) (Invitrogen, 1:5000), for 5 min in 1X PBS, and then washed twice with 1X PBS for 5 min before mounting with a home-made PVA-DABCO-based mounting medium (Glycerol (Sigma 49782-1L, 24%), PVA (Sigma P8136-250g, 9.6%), 96mMTrisHCl, DABCO (Sigma D27802-100G, 2.5%)).

LDs in brain sections were visualized using the same lipophilic dyes. Briefly, brain sections were initially washed twice for 5 min each in 1X PBS. Subsequently, they were incubated for 2 h at RT on an orbital shaker with the different dyes and DAPI simultaneously (except for LipiDyeII, which is also excited by the 405nm laser, and thus cannot be combined with DAPI), using the concentrations indicated above. Following the incubation with the fluorescent dyes and DAPI, the sections were washed twice in 1X PBS for 5 min and were then mounted with a home-made PVA-DABCO-based mounting medium.

#### Immunohistochemistry of PLIN2 with LD fluorescent dyes

For immunohistochemical analysis of PLIN2 and fluorescent dyes, sections were washed three times in 1X PBS for 5 min on an orbital shaker at RT. Then, sections were permeabilized for 1 h in 1X PBS containing 0.3% or 0.15% Triton X-100 and 10% donkey serum (#S30-100mL, Merck). Thereafter, they were immunolabeled for 48 h at 4°C on an orbital shaker, using the following primary antibodies: Rabbit-PLIN2 (1:600, #ab52356, Abcam). Following the primary antibody incubation, the sections underwent three additional washes in 1X PBS for 5 min each and were then incubated for 2 h at RT with fluorescent secondary antibodies (AlexaFluor, anti-rabbit 488, donkey anti-rabbit 594, Jackson, 711-545-152) diluted in 1X PBS. Finally, nuclei and LDs were counterstained with DAPI and the following fluorescent dyes: BODIPY 493/503 4,4-DIFLUORO-1,3,5,7,8-PE (Fisher Scientific 11540326, dilution 1:1000 in 1X PBS); LipidSpot488 (VWR 70065-T, dilution 1:1000 in 1X PBS); LipidSpot610 (VWR 70069-T, dilution 1:1000 in 1X PBS); LipiDyeII 488 (Anawa, FDV-0027, dilution 1:1000 in 1X PBS). After 2 h of incubation, the sections were washed twice in 1X PBS and then mounted with a home-made PVA-DABCO-based mounting medium.

#### Confocal microscopy acquisition and image analysis

All images were collected on a Leica confocal imaging system (TCS SP8) with 63× (0.75 and 2 NA) oil immersion objective. For the quantification of LDs, serial sections of the cortex and SVZ were used. Briefly, Z-stacks were taken at 0.3 μm intervals, and the number and the diameter of LDs in the 8-week-old and 2-year-old mouse brain were measured using IMARIS software. The “Surface” function was used to perform a 3D reconstruction of all LDs, with a minimum volume threshold set at 0.05 μm^3^. To account for different number of cells, the percentage of area covered was normalized to the number of nuclei stained with DAPI. Of note, the number of nuclei when LDs were stained with LipiDyeII were counted as depicted in Figure S2A, by overexposing the background. Three images per sections and several sections per regions were taken from 3 to 4 mice per group. To quantify the colocalization of LipidSpot488 with the tdTom-PLIN2 signal in mouse brain section, 3 images per section were taken using 63× magnification with a digital zoom 2x. The quantification of colocalized LDs was performed manually using the multipoint tool in Fiji.

For the quantification of colocalization of Lipid Dyes with tdTom-Plin2 in NSPCs, 3 coverslips per condition were stained, 3 images per coverslip were taken using digital zoom 3x and all Lipid Droplets in the field of view were analyzed using IMARIS software. Briefly, “Spots” function was created for lipid droplets stained with Lipid Dyes and for tdTom-Plin2 positive lipid droplets. Colocalization between two created spot sets was analyzed using “Spot Colocalization” function.

For the quantification of colocalization of endosomal markers with the tdTom-PLIN2 signal in NSPCs, 3 coverslips per condition were stained, 5 images per coverslip were taken using a 63× objective and a digital zoom 2x, and all LDs and endosomal structures in the field of view were analyzed using IMARIS software. Briefly, a surface using the “Surface” function was created for the endosomal structures stained with endosomal markers and for tdTom-PLIN2 positive LDs. A % of colocalization between the two created surfaces was calculated with IMARIS by dividing the volume of endosomal structures that overlap with tdTom-PLIN2 volume by total tdTom-PLIN2 volume.

#### Illustration software

For illustration schemes, Biorender software (Biorender, 2021), Adobe Illustrator (version 25.0, Adobe Inc) and Affinity Designer 2 (version 2.5.2, Eula) were used.

### Quantification and statistical analysis

To compare the data obtained from the two hemispheres undergoing different tissue sectioning, paired *t* test were used. For comparing the influence of tissue sectioning and detergent use, a two-way ANOVA followed by Fishers LSD test was used. For comparing the LD numbers in WT and tdTom-Plin2 unstained and stained tissue, an ordinary one-way ANOVA was performed. For comparing the LD numbers in WT and tdTom-Plin2 unstained and stained tissue, an ordinary one-way ANOVA was performed. For frequency distribution comparison data were transformed using arcsin(sqrt(Y)) followed by an ordinary two-way ANOVA with age and LD size distribution as factors.

All analyses were performed using GraphPad Prism 10.1.2 software (GraphPad software). Each figure legend contains the statistical details of the experiments, including the statistical tests used, exact value of n, what n represents, and which measure is shown.

## Supplemental information

Document S1. Figures S1–S6

Document S2. Article plus supplemental information
