# Supplementary material for: An optimized method to visualize lipid droplets in mouse brain tissue
Source: Cell Rep Methods. 2026 May 13;6(6):101455. doi: 10.1016/j.crmeth.2026.101455 (PMC13282654; doi:10.1016/j.crmeth.2026.101455)
Supplement: Document S1. Figures S1–S6 [file mmc1.pdf]

Cell Reports Methods, Volume *6*

## Supplemental information

# An optimized method to visualize lipid droplets in mouse brain tissue

**Alicia Rey, Francesco Petrelli, Diana Panfilova, Sofia Madsen, Noéline Héritier, and Marlen Knobloch**

# Supplementary Figure 1

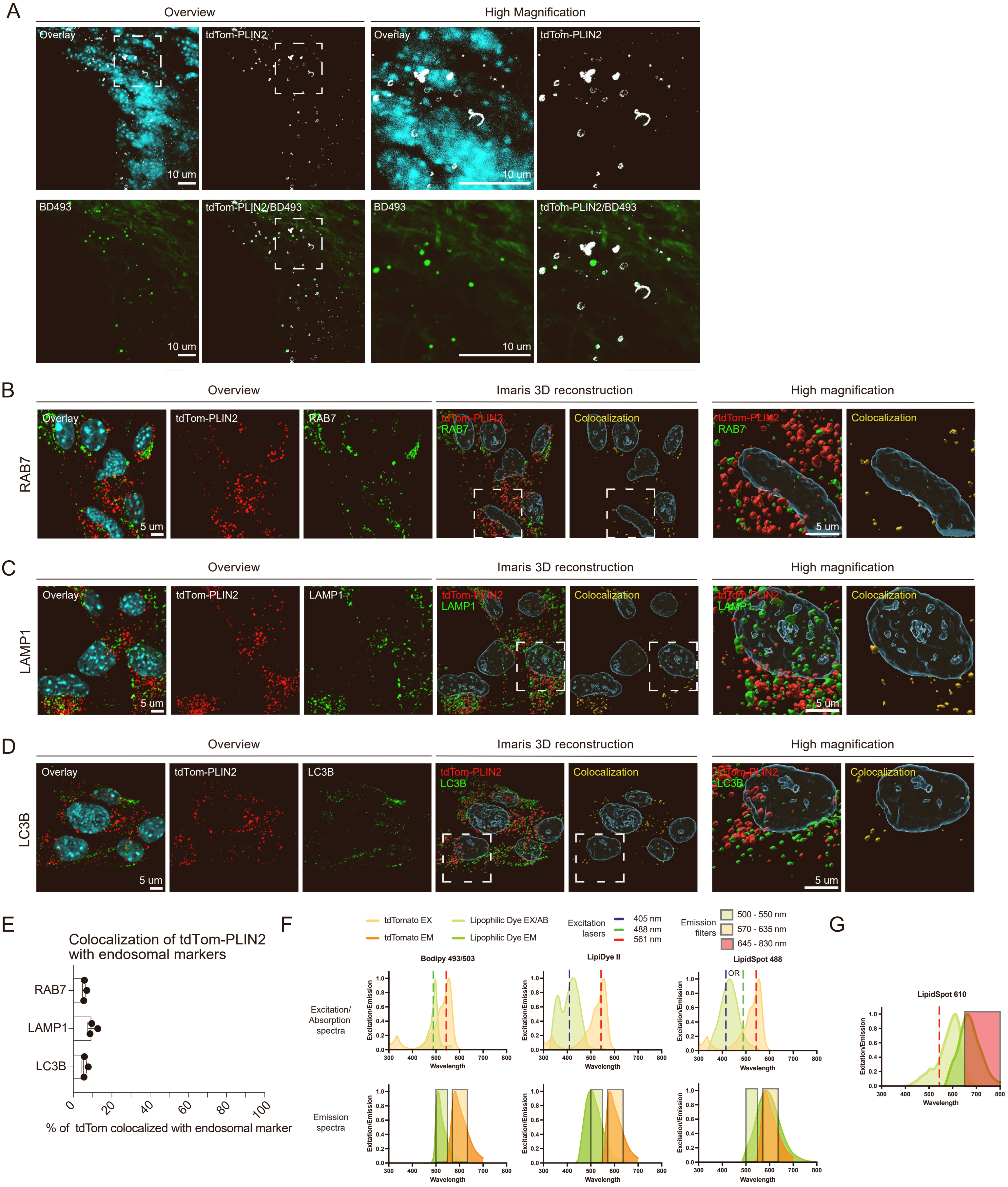

**Supplementary Figure 1: BD493 works in cells but does not reveal LDs in young adult mouse brain tissue, related to Figure 1.**

**A)** Representative overview and high magnification confocal images (maximum projections) display tdTomato (tdTom-PLIN2, in white), BODIPY 493/503 (BD493, in green) and DAPI (cyan) in the subventricular zone (SVZ) of 8-week-old tdTom-Plin2 mice. **B-D)** Representative overview of confocal images (maximum projections) and their 3D reconstruction using IMARIS, to assess colocalization with 3 different markers of the endosomal/lysosomal pathway, such as RAB7 (B, green), LAMP1 (C, green), and LC3 (D, green). DAPI (cyan) and TdTom-PLIN2 (red). The colocalization between tdTom-PLIN2 and the endosomal/lysosomal markers is highlighted in yellow. **E)** Quantification of the % colocalization for RAB7, LAMP1 and LC3 shows that only a small % of the tdTom-PLIN2 signal is coming from the endosomal/lysosomal degradation pathways. Each dot represents data from a separate coverslip, with n=3 coverslips per group. The data represent the mean value +/- SEM. **F and G)** Graphs illustrate the excitation and absorption spectra of BODIPY 493/503, LipiDyeII, LipidSpot488 and LipidSpot610. The spectra for tdTomato, BD493/503, LipidSpot488 and LipidSpot610 were generated using fpbase.org. The spectra for LipiDyeII were adapted from data provided by https://www.diagnocine.com/Product/LipiDye-II-Lipid-dye-Droplet-Staining/67496. Note that LipidSpot610 cannot be used together with tdTomato due to the spectral overlap, and that LipiDyeII is also excited by the 405nm laser, thus cannot be combined with DAPI. Scale bars: 10µm (A), 5µm (B-D).

# Supplementary Figure 2

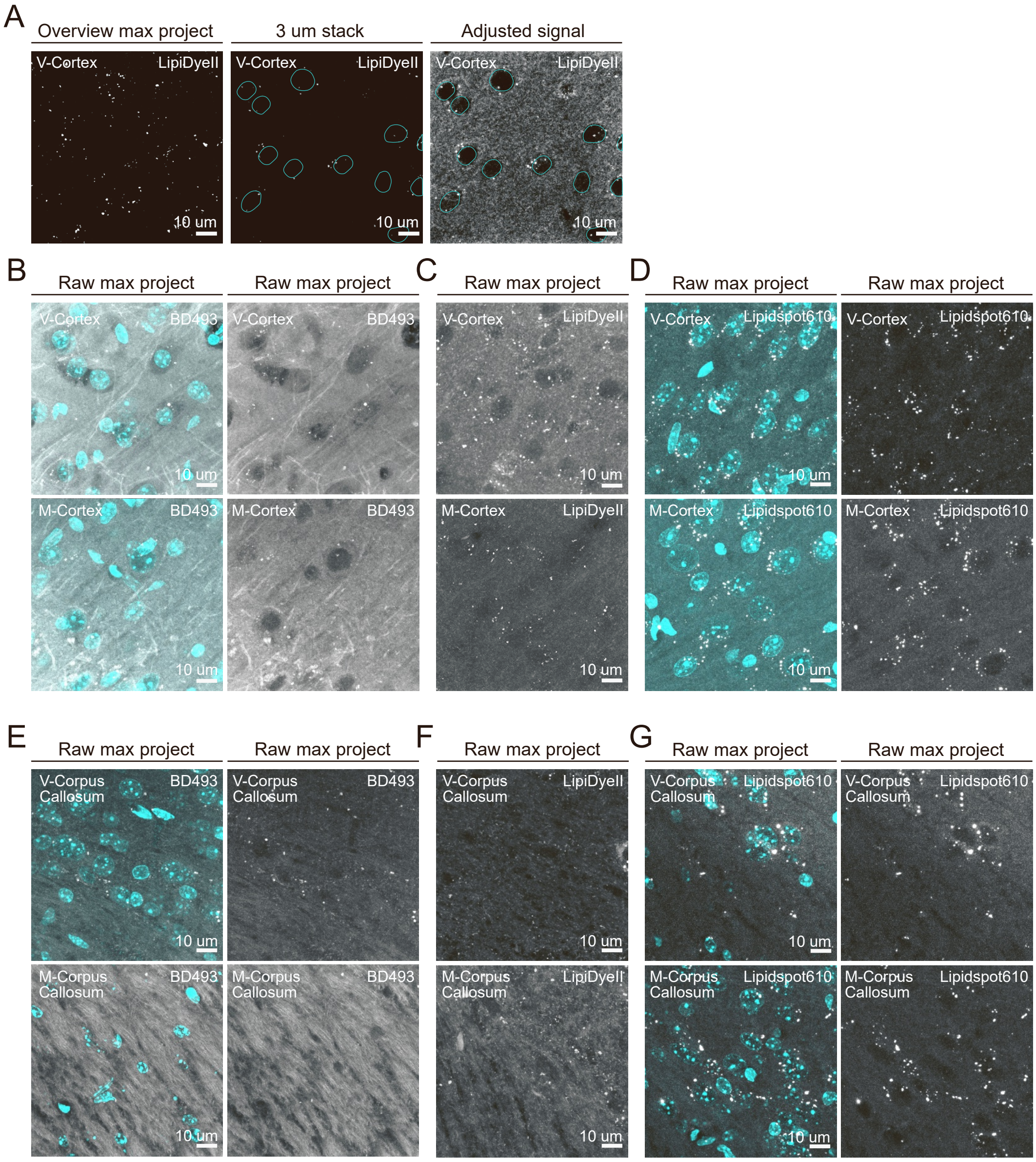


**Supplementary Figure 2: Alternative lipophilic dyes detect a large number of LDs *in vitro* and in brain tissue, related to Figure 2.**

**A)** Representative confocal images show an overview (maximum projection), a 3 µm projection and the adjusted signal of LipiDyeII (white) staining in vibratome sagittal cortical sections of 8-week-old WT mice, to illustrate how the signal can be used to determine cell nuclei. Due to the excitation spectra, DAPI cannot be used. **B-D)** Representative confocal images show the raw maximum projections of BD493 (white) or LipiDyeII (white) or LipidSpot610 (white) and DAPI (cyan) staining in microtome (M) and vibratome (V)-derived cortical sections of WT mice. **E-F)** Representative confocal images show the raw maximum projections of BD493 (white) or LipiDyeII (white) or LipidSpot610 (white) and DAPI (cyan) staining in microtome (M) and vibratome (V)-derived corpus callosum sections of WT mice. Scale bars for all images: 10µm.

# Supplementary Figure 3

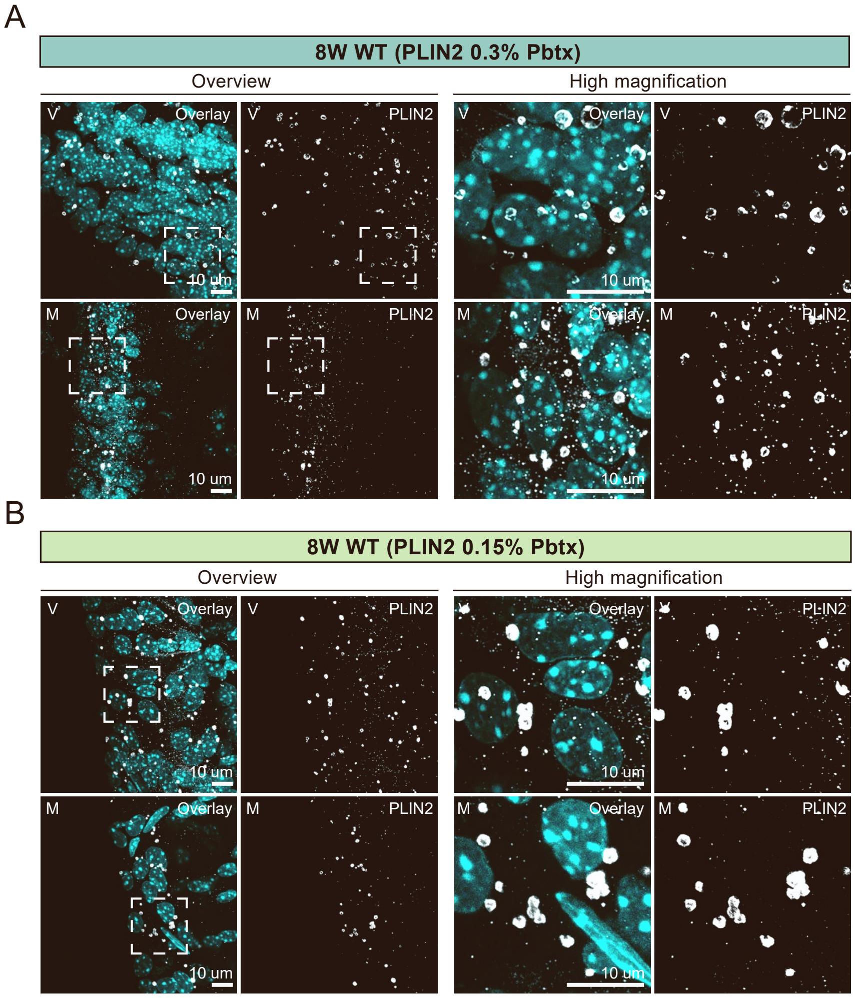


**Supplementary Figure 3: Staining outcome using a PLIN2 antibody in young adult mouse brain tissue depends on tissue treatment, related to Figure 3.**
**A and B)** Representative overview and high magnification confocal images (maximum projections showing PLIN2 (white) and DAPI (cyan) immunostaining with 0.3% or 0.15% Pbtx in microtome (M) and vibratome (V)-derived SVZ sections of WT mice. Scale bars: 10µm.

# Supplementary Figure 4

A

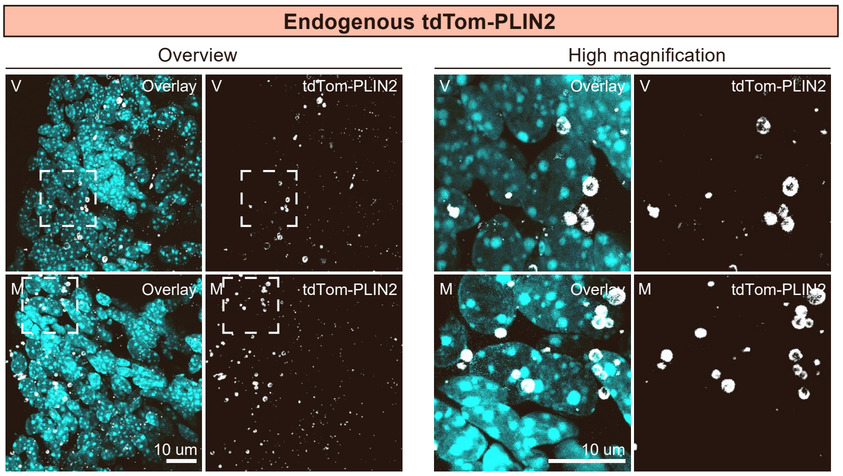


B

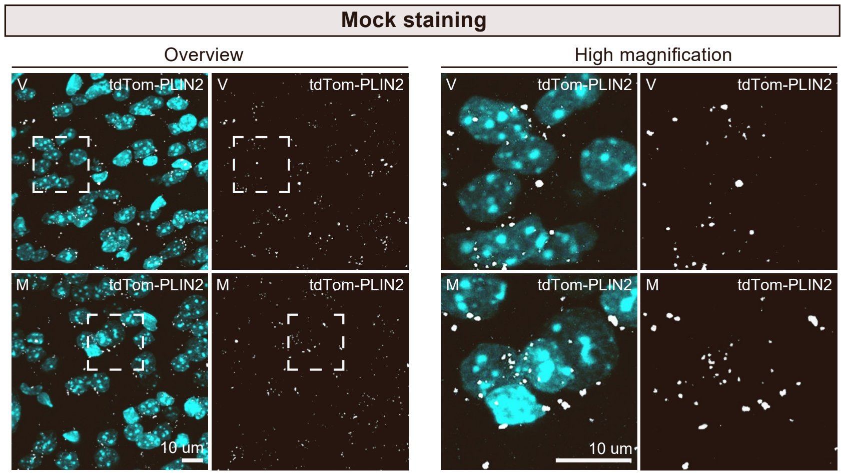


C

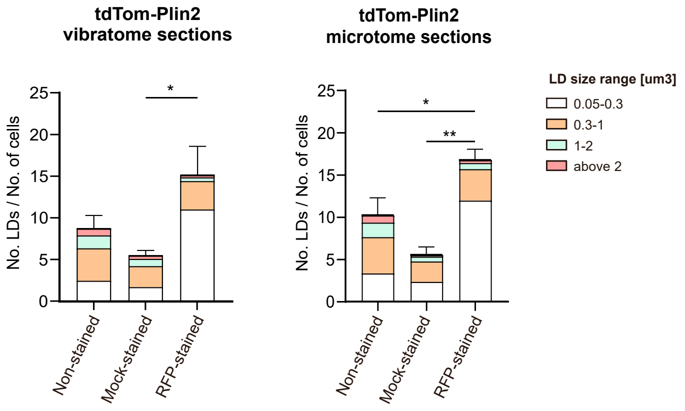


D

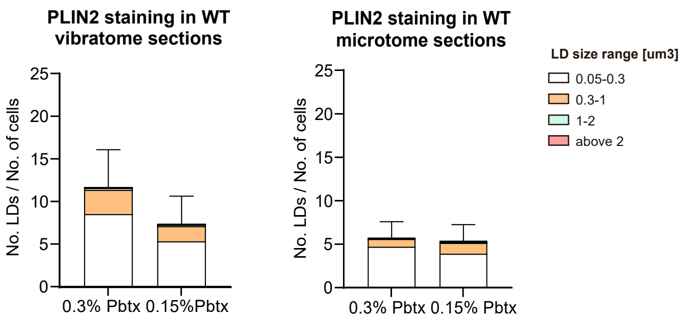

**Supplementary Figure 4: Endogenous tdTom-PLIN2 signal does not depend on the tissue sectioning method, related to Figure 4.**

**A)** Representative overview and high magnification confocal images (maximum projections) showing tdTomato (tdTom-PLIN2, in white) and DAPI (cyan) in microtome (M) and vibratome (V)-derived SVZ sections of tdTom-Plin2 mice. **B)** Representative overview and high magnification confocal images (maximum projections) showing tdTomato (tdTom-PLIN2, in white) and DAPI (cyan) in microtome (M) and vibratome (V)-derived cortex sections of tdTom-Plin2 mice, which underwent a mock-staining procedure (incubating sections with a staining solution that did not contain an antibody). **C and D)** Superimposed bar charts show the total number of LDs and their respective size distribution, ranging from 0.05 $\mu m^3$ to above 2 $\mu m^3$, across the different experimental conditions (non-stained, mock-stained, RFP-stained or 0.3% Pbtx, 0.15% Pbtx) in sections from tdTom-Plin2 mice (C) and in sections from WT mice (D). *n*= 3 mice per condition. SEM is depicted for the total number of LDs. One-way ANOVA for total number of LDs, p-value: *<0.05, **<0.01. Scale bars: 10µm (A and B).

# Supplementary Figure 5

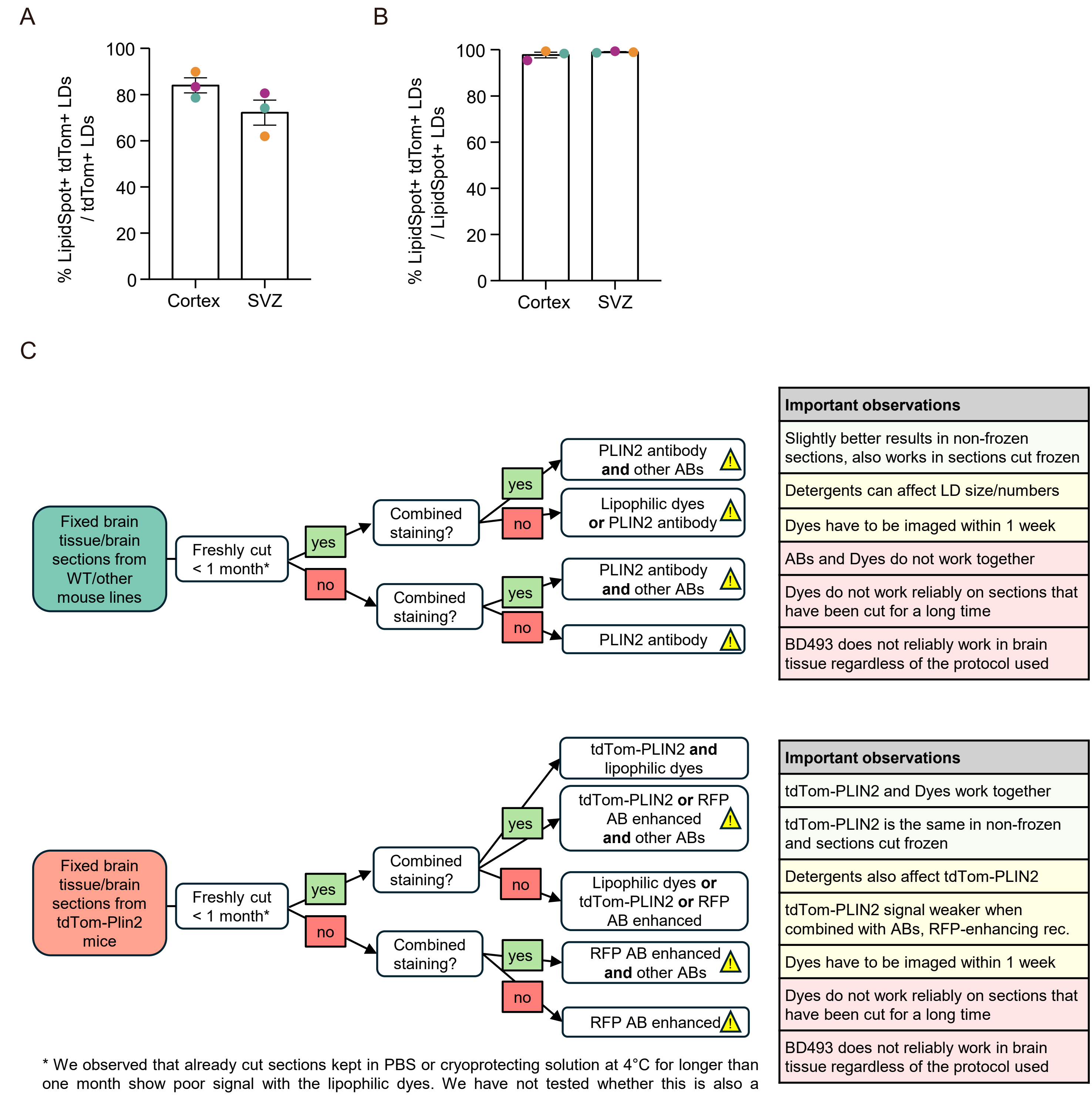


* We observed that already cut sections kept in PBS or cryoprotecting solution at 4°C for longer than one month show poor signal with the lipophilic dyes. We have not tested whether this is also a problem when kept at -20°C. Fixed, non-cut brains can be kept for a very long time at 4°C without a loss in dye signals when freshly cut afterwards. AB staining is not affected by these storage factors.

**Supplementary Figure 5: Simultaneous detection of LD core and LD coat works in tdTom-Plin2 brain sections, related to Figure 5**
**A and B)** Quantification of the colocalization of LipidSpot488 and tdTom-PLIN2 in cortex and SVZ sections from tdTom-Plin2 mice. The colocalization % is either normalized to all tdTom-PLIN2 positive structures (A) or to all the LipidSpot488 positive structures (B). **C)** Flow chart depicting the best staining procedure, depending on the starting material and the desired analysis, as well as a list of important observations regarding the different procedures. Each method has its advantages and disadvantages.

Supplementary Figure 6

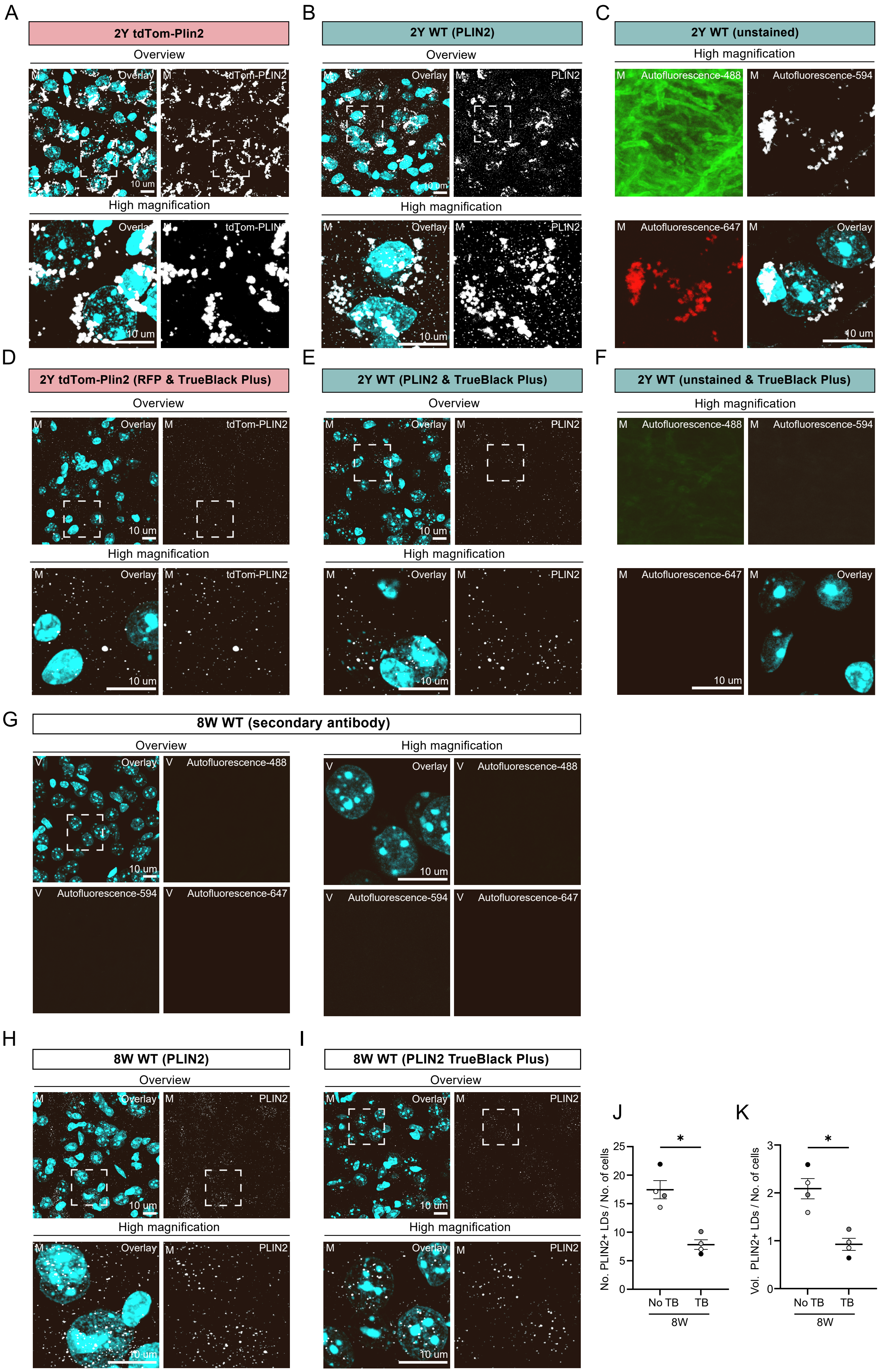

**Supplementary Figure 6: The number of LDs in the brains of 2-year-old mice are variable, with larger sizes compared to young mice, related to Figure 6**

**A)** Overview and high magnification confocal images (maximum projections) showing tdTomato (tdTom-PLIN2, white) and DAPI (cyan) in the cortex of 2-year-old tdTom-Plin2 mouse. **B)** Overview and high magnification confocal images (maximum projections) showing PLIN2 (white) and DAPI (cyan) in the cortex of 2-year-old WT mouse. **C)** High magnification confocal image (maximum projection) showing individual imaging channels: 488-nm laser (green), 594-nm laser (white), and 647-nm laser (red) and DAPI (cyan) in the cortex of an unstained 2-year-old WT mouse **D)** Overview and high magnification confocal images (maximum projections) showing tdTomato (tdTom-PLIN2, white) and DAPI (cyan) in the cortex of a 2-year-old tdTom-Plin2 mouse after a 5-minute treatment with TrueBlack Plus. **E)** Overview and high magnification confocal images (maximum projections) showing PLIN2 (white) and DAPI (cyan) in the cortex of a 2-year-old WT mouse after a 5-minute treatment with TrueBlack Plus. **F)** High magnification confocal image (maximum projection) showing individual imaging channels: 488-nm laser (green), 594-nm laser (white), and 647-nm laser (red) and DAPI (cyan) in the cortex of an unstained 2-year-old WT mouse after a 5-minute treatment with TrueBlack Plus. **G**) Overview and high magnification confocal (maximum projections) image showing individual imaging channels: 488-nm laser (green), 594-nm laser (white), and 647-nm laser (red) and DAPI (cyan) in the unstained cortex of a 8-week-old WT mouse. **H and I)** Overview and high magnification confocal images (maximum projections) showing PLIN2 (white) and DAPI (cyan) in the cortex of a 8-week-old WT mouse without (H) and with (I) a 5-minute treatment with TrueBlack Plus. **J and K)** Quantification of the total number (J) and total volume (K) of PLIN2 positive LDs per number of cells in 8-week-old mice without and with TrueBlack Plus. Each dot represents an individual mouse, with n=4 mice per group. The data represent the mean value +/- SEM. Unpaired Student t-test. *p* value *<0.05. Scale bars for all images: 10µm.
